# Seed Paternity Analysis Using SSR Markers to Assess Successful Pollen Donors in Mixed Olive Orchards

**DOI:** 10.3390/plants10112356

**Published:** 2021-10-31

**Authors:** Gabriela Vuletin Selak, Alenka Baruca Arbeiter, Julián Cuevas, Slavko Perica, Petar Pujic, Marina Raboteg Božiković, Dunja Bandelj

**Affiliations:** 1 Department of Plant Sciences, Institute for Adriatic Crops and Karst Reclamation, 21000 Split, Croatia; Slavko.Perica@krs.hr (S.P.); marina.raboteg@krs.hr (M.R.B.); 2 Centre of Excellence for Biodiversity and Molecular Plant Breeding (CoE CroP-BioDiv), Svetošimunska Cesta 25, 10000 Zagreb, Croatia; 3 Faculty of Mathematics, Natural Sciences and Information Technologies, University of Primorska, 6000 Koper, Slovenia; alenka.arbeiter@upr.si (A.B.A.); dunja.bandelj@famnit.upr.si (D.B.); 4 Department of Agronomy, University of Almería, CeiA3, La Cañada de San Urbano, s/n, 04120 Almería, Spain; jcuevas@ual.es; 5 Univ Lyon, Université Claude Bernard Lyon 1, CNRS, INRAE, VetAgro Sup, UMR5557 Ecologie Microbienne, F-69622 Villeurbanne, France; petar.pujic@univ-lyon1.fr

**Keywords:** *Olea europaea* L., cultivar ‘Oblica’, microsatellites, seed paternity assignment, cross-compatibility, self-incompatibility

## Abstract

The olive tree (*Olea europaea* L.) is a wind-pollinated crop that exhibits an extreme alternate bearing habit. To improve fruit set, several methods have been used to determine the most successful compatible combinations of cultivars. In this study, priority is given to seed paternity analysis based on simple sequence repeats (SSRs), microsatellite markers used for the identification of potential pollen donors of cultivar ‘Oblica’ in a mixed olive orchard during two consecutive years. Seven microsatellite primers were successfully used to examine the paternity of olive embryos from ‘Oblica’ mother trees. Embryos were considered as a product of self-fertilization if only maternal alleles were present, but not a single case of self-fertilization was found among all the embryos analyzed. Two dominant pollen donors were not the closest nor the cultivars with the highest number of trees in the orchard, suggesting that cross-compatibility may have a key role in determining pollen donor success. In our earlier studies, pollen tube growth and fertilization success correlated with fruit set when controlled crosses between cultivars were performed; however, some discrepancy might appear compared to paternity analyses when mother trees have a free choice among different pollen sources from cultivars growing in their surroundings.

## 1. Introduction

The olive, *Olea europaea* L., is a wind-pollinated, hermaphrodite, preferentially allogamous crop extraordinarily important in the Mediterranean area. Olive presents abundant biennial flowering, although a relative poor fruit set, even in its ‘off’ season [1]. The constraints leading to low fruit set include alternate bearing [2], male-sterility [3], pistil abortion [4,5], and self- (SI) and cross-incompatibility [6,7]. However, SI is certainly the most important reproductive barrier in olive. SI prevents self-fertilization based on mechanisms involving the recognition and rejection of self-pollen [8,9]. Cross-fertilization increases genetic variability and consequently imparts strong evolutionary potential. A higher success of cross-pollination over self-pollination has been generally reported for olive and the self-incompatible condition of olive is no longer disputed [10,11,12,13].

The genetic of SI is, however, a controversial issue in olive since there are discussions about the type of SI olive presents [14,15]. Olive has been classified as a species having gametophytic SI (GSI), mostly based on morphological traits, such as wet-type stigma and binucleate pollen grains at the moment pollen is released [16]. According to this model, combinations of two cultivars will give similar results for crosses in both directions. However, this model failed to explain fruit set obtained in earlier studies, thus a sporophytic self-incompatibility (SSI) system was proposed [15,16,17,18,19,20]. Recently, a diallelic self-incompatibility (DSI) system based on observations of reciprocal pollen–pistil interactions led Saumitou-Laprade et al. [21] and Mariotti et al. [22] to conclude that in olive there are only two incompatibility groups where cultivars are incompatible within groups and compatible between groups.

SI in tree crops obliges growers to plant more than one cultivar in their orchard in order to obtain a good yield. The knowledge of cross-compatibility relationships in olive is important for orchard designs, since in some cases, cross-incompatibility reactions occurs, as it is the situation between ‘Manzanilla de Sevilla’ and ‘Mission’ [23]. Therefore, the choice of cultivars and their distribution within the orchard will determine to a great extent the fertilization success, fruit set levels, and consequently yields, having, however, in mind wind as the pollination vector in olive and its capacity to disperse pollen grains to large distances.

Different methods have been used to identify compatible cross combinations in olive: observation of pollen tube growth in response to selected cross-pollination treatments [24,25] and/or measurements of resulting fruit set after hand cross-pollination [11,12,23]. A different approach is seed paternity analysis facilitated by molecular markers that identify inherited variations among seedling genotypes. Among the various markers utilized in paternity analysis, including Single Nucleotide Polymorphisms (SNPs), Amplified Fragment Length Polymorphisms (AFLPs), and Diversity Array Technology (DArT) markers [26], microsatellites or Simple Sequence Repeats (SSRs) were proved useful for seed paternity and kinship analyses [22,27,28,29,30,31,32]. Paternity analysis using SSR markers involves DNA profiling of known maternal parent, potential paternal parents (pollen donors), and offspring. Then, the information obtained on the genotype profile is used to assign the progeny to the correct parental pair. FaMoz software for parentage studies based on microsatellite data has been demonstrated in olive [28,30,33,34,35,36], grape [37,38,39], apple [40], and blueberry [41]. The identification of the paternal parent using SSR markers is proposed as a reliable method for pollination studies in olive because the genetic contribution of alleles is traced from the parents to the offspring [22,27,28,29,30,31,32]. Microsatellites are suitable for this purpose owing to their codominant inheritance and high polymorphism in olive [42,43]. Paternity analysis can also be used to assess self-incompatibility response as was shown for ‘Kalamata’ [44], ‘Arbequina’, ‘Picual’ [29], and different Italian olive cultivars [31]. DNA fingerprinting and paternity analyses extend their utility to olive breeding programs [27], since the testing of the parentage of the progeny verifies the cross as well as the compatibility between parent cultivars. In breeding programs, the seeds are germinated and DNA isolated from true leaves of the seedlings according to the procedure described by De la Rosa et al. [27]. In pollination experiments, the DNA is extracted directly from uncoated seeds as described by Diaz et al. [29]. In different studies, two [31], four [27,45], seven [30], or eight [28,44] microsatellites have been used for the identification of the genotypes acting as parents of the embryos or seedlings.

‘Oblica’ is the most widespread olive cultivar in Croatia and is used for oil and table olive production. In old monovarietal groves, ‘Oblica’ produces low yields. However, in newly established orchards, in the presence of other cultivars, fruit set and yield increase. The determination of successful, cross-compatible combinations of olive cultivars in Croatia, was our highest interest in earlier studies [25,46], especially regarding compatibility with newly introduced foreign cultivars. In those studies, after controlled cross-pollination, pollen tube growth and fruit set were measured to assess the compatibility between cultivars. The aim of the present work was to determine the most efficient pollen donors for olive cultivar ‘Oblica’ and the proportion of self-fertilization in a multivarietal olive grove using microsatellite markers for seed paternity analyses. We were especially interested in knowing the efficiency of different paternal parents to contribute to successful fertilization when mother trees had a free choice of pollen donor sources.

## 2. Materials and Methods

### 2.1. Plant Material for Paternity Analysis

**Figure 1:**
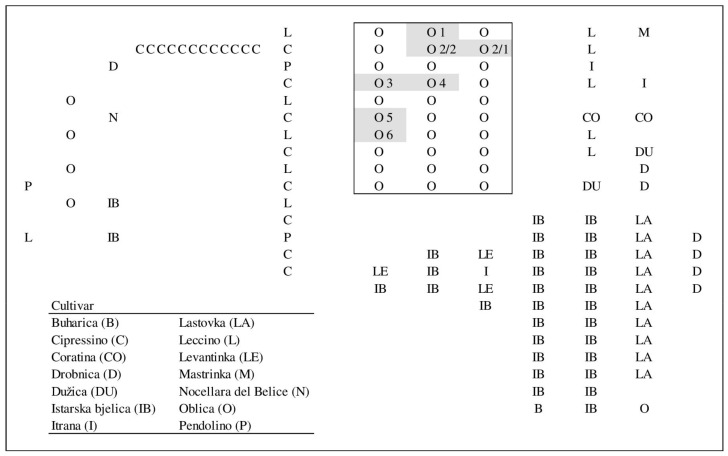
Orchard design showing the position of potential pollen donor cultivars and selected mother trees (indicated as O1–O6; O2/1 is mother tree 2 selected in 2017 and O2/2 is mother tree 2 selected in 2018).

The study was conducted in a mixed olive orchard in Kaštela (43°54′94″ N, 16°29′95″ E), Split-Dalmatia County, Croatia, during 2017 and 2018 (Figure 1). The cultivars present in the orchard were ‘Buharica’, ‘Cipressino’, ‘Coratina’, ‘Drobnica’, ‘Dužica’, ‘Istarska bjelica’, ‘Itrana’, ‘Lastovka’, ‘Leccino’, ‘Levantinka’, ‘Mastrinka’, ‘Nocellara del Belice’, ‘Oblica’, and ‘Pendolino’. ‘Oblica’, ‘Levantinka’, and ‘Leccino’ are the mostly widely planted cultivars throughout the olive-growing area in Croatia, while ‘Istarska bjelica’ is most widely planted in Istria. The cultivars were represented in the orchard by a different number of trees (Figure 1). We considered all cultivars as potential pollen donors, and for this reason, young leaves were collected for genotyping all fourteen cultivars at the beginning of the experimentation.

For paternity analyses, six (in 2017) and five (in 2018) trees of cultivar ‘Oblica’ were selected for their high level of fruit load and denoted as mother trees. At least sixty fruits per mother tree were collected. Fruits were harvested across the canopy segments facing each direction (north, south, east, and west), making in total 622 fruits (embryos) examined over the two years of the trial. The flowering periods of the cultivars were assessed twice per week by following the phenology of the trees present in the orchard according to Barranco et al. [47], in both years. The weather conditions, daily mean temperatures and wind speed and direction, during the experiment were registered at meteorological station near the orchard. The orchard was managed following standard commercial practices. 

### 2.2. Extraction of High-Quality DNA Using Modified Protocols

Freshly collected leaves from representative trees of the different genotypes present in the orchard and acting as potential pollen donors, together with leaves from selected mother trees (‘Oblica’), were transferred to the laboratory and stored at 4 °C until DNA extraction was carried out the next day. Total DNA from leaf material was extracted using the slightly modified Cetyl Trimethyl Ammonium Bromide–PolyVinylPyrrolidone (CTAB–PVP) protocol developed by Japelaghi et al. [48], with some modifications reported by Miklavčič Višnjevec et al. [49].

**Figure 2:**
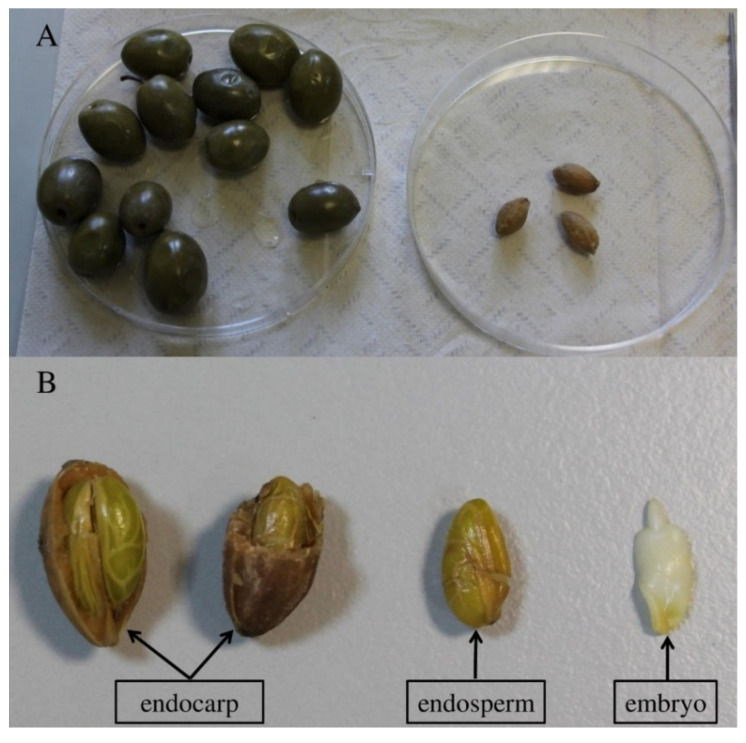
Olive fruit before and after removal of exocarp and mesocarp (**A**). Broken endocarp, endosperm, and embryo visible after dissection of endosperm (**B**).

To obtain the embryo for DNA extraction, the exocarp and mesocarp were removed and the endocarp cracked (Figure 2). The diploid embryo was separated from the endosperm using a scalpel.

The DNA extraction from the embryos was performed according to the modified method developed by Guerin and Sedgley [50]. Each single embryo was immersed in 500 µL of grinding buffer (100 mM Tris, pH 8.0, 20 mM EDTA, pH 8.0, with 4 mg/mL diethyl dithiocarbamic acid sodium salt added just before use) in a 2 mL microcentrifuge tube. The embryo was ground with the buffer and kept on ice until all the samples were prepared. The samples were incubated for 10 min at 65 °C, followed by the addition of 500 µL of lysis buffer (100 mM Tris, pH 8.0, 20 mM EDTA, pH 8.0, 1 M NaCl, 2% (*w/v*) SDS, and 1% (*w/v*) sodium metabisulphite added just before use) and further incubated for 30 min at 65 °C. Samples were cooled on ice and an equal volume (1 mL) of cold phenol:chloroform:isoamyl alcohol (25:24:1) was added and mixed. The samples were centrifuged for 20 min at 14,000× *g* rpm and the supernatant was removed to 1.5 mL centrifuge tube. The DNA was precipitated using 500 µL of ice-cold isopropanol. The samples were kept in a freezer for 1.5 h and then centrifuged for 15 min at 14,000× *g* rpm. The supernatant was removed. The pellets were washed in 1 mL of 75% ethanol. The supernatant was decanted and the DNA pellets dried at room temperature. Pellets were then dissolved in 50 µL of TE buffer (10 mM Tris–HCl, 1 mM EDTA, pH 8.0). In order to obtain a high quality and sufficient concentration of DNA and to improve the PCR amplification, the method used for extraction of DNA from embryos was modified [50]. The main modification was the repeated extraction using the protocol CTAB–PVP, appropriate for DNA isolation from plant tissues rich in polyphenols and polysaccharide compounds [48]. Previously dissolved DNA samples extracted from the embryos were purified by using 400 µL of CTAB–PVP extraction buffer (2% (*w*/*v*) CTAB, 2% (*w*/*v*) soluble PVP, 2 M NaCl, 25 mM EDTA (pH 8.0), 300 mM Tris–HCl (pH 8.0), 2% (*w/v*) β-mercaptoethanol). The samples were incubated for 10 min at 65 °C, followed by the addition of 200 µL of phenol:chloroform:isoamyl alcohol (25:24:1). The samples were mixed and centrifuged for 10 min at 14,000× *g* rpm and the supernatant was removed to 1.5 mL centrifuge tube. The step with phenol:chloroform isoamyl alcohol was repeated once again and then the DNA was precipitated using 30 µL of 3 mM sodium acetate and 300 µL of ice-cold isopropanol. The samples were kept in a freezer at −80 °C for 30 min and then centrifuged for 30 min at 14,000× *g* rpm. The supernatant was removed. The pellets were washed in 500 µL of 70% ethanol, dried at room temperature, and finally dissolved in 30 µL of TE buffer (10 mM Tris–HCl, 1 mM EDTA, pH 8.0). The optimized CTAB–PVP protocol enabled the extraction of high quality genomic DNA, since the amplification success was significantly improved, especially for samples with extremely low DNA yields (<5 ng).

Finally, the DNA concentration of potential pollen donors was quantified using the Qubit^TM^ 1.0 fluorometer (Thermo Fisher Scientific, Waltham, MA, USA) and the DNA diluted to 10 ng/μL. However, the DNA from the embryos was not quantified and consequently not diluted due to their extremely low DNA concentrations (in a range from 5 to 30 ng/μL).

### 2.3. Genotyping Procedure

The samples (olive embryos and leaves from ‘Oblica’ trees and leaves from potential pollen donors) were characterized by seven microsatellite loci: ssrOeUA-DCA-(3, 9, 11, 16) [42], GAPU101 [51], EMO3 [43], and UDO99–019 [52] (Supplementary Table S1). PCR amplification was carried out using DNA Engine Thermal Cycler 200 (Bio-Rad Laboratories, CA, USA) in a 15 µL reaction volume containing 1× supplied PCR buffer; 2 mM MgCl_2_; dNTPs (0.2 mM of each dNTP) (Promega); 1.25 units of Taq DNA polymerase (Promega); 0.2 µM of each locus specific primer (synthesized by IDT); 0.25 µM of a third universal primer M13(−21) [53] labeled with a fluorescent dye 6-FAM, VIC, PET, or NED (Applied Biosystems); and 40 ng of template DNA for potential pollen donors or 4 µL of undiluted template DNA for olive embryos. The two-step touch-down amplification profile consisted of an initial denaturation at 94 °C for 5 min, followed by 5 cycles at 94 °C for 30 s, 57 °C for 30 s, and 72 °C for 30 s, where the annealing temperature was reduced by 1 °C per cycle, then followed by 30 cycles of 94 °C for 30 s, 52 °C for 30 s, and 72 °C for 30 s. The final extension step was carried out at 72 °C for 8 min. The PCR products were separated on an SeqStudio^TM^ Genetic Analyzer (Applied Biosystems, Waltham, MA, USA) and allele lengths were determined using GeneMapper software, version 4.1 (Applied Biosystems, Waltham, MA, USA).

### 2.4. Data Analysis

For the potential paternal parents, the following genetic parameters for each of the seven microsatellite loci were calculated: polymorphic information content (PIC), probability of identity (PI), and exclusion probability (EP). The CERVUS 3.0.7 program [54] was used to calculate PIC, while PI and EP were computed with program FaMoz [33] (http://www.pierroton.inra.fr/genetics/labo/Software/Famoz/index.html, accessed on 1 April 2019).

Potential pollen donors for the olive variety ‘Oblica’ were identified using paternity analyses assigning each genetically known mother–offspring pair to its most likely father. The probability of exclusion [55], probability of identity [56], and LOD scores (log of the odds ratio or likelihood ratio for potential parent–offspring relationships) were calculated using the program FaMoz [33]. The 1000 simulated offspring from the genotyped parents were performed to determine LOD score threshold for assessing a true pollen donor with microsatellite markers. The LOD score threshold was determined by comparing the curves of two simulations. The first simulation was done on 1000 randomly generated offspring with father randomly chosen among the 14 genotyped parents. The second simulation was performed on 1000 offspring whose paternal genotype was randomly generated according to allele frequencies in the parental population. The threshold was chosen at the intersection of the two distributions of LOD scores. Only parent–offspring pairs with LOD scores above the threshold value were considered. The genotyping error in the simulation and in the assignment of the most likely pollen donor was set to 1% [33], due to possible errors which could occur in the phase of allele calling.

The relationships between seed fathering success and the abundance of trees of each potential pollinizer and with the distance to them was explored by correlation and regression analyses. In the analyses, we considered the distance in meters as the distance of the mother trees (those providing the embryos for analyses) to the closest tree of each potential pollen donor. Although not fully accurate, we chose central ‘Oblica’ tree for the calculation of the distance between mother trees and pollen donor trees. Since the proportion of potential pollinizers in the experimental orchard differed and this could be an additional influential factor, we explored the relation between their abundance (also taking into account their canopy volume) and the number of embryo fathered considering the number of trees of each cultivar present in the orchard.

## 3. Results

For paternity analysis of a large number of samples it is essential to extract high quality DNA. Olive embryos accumulate high contents of polysaccharides, polyphenols, and other secondary metabolites [57,58]. These compounds tend to bind and/or coprecipitate with DNA, interfering with the PCR performance [59]. Therefore, a stable and highly throughput DNA extraction method has been developed in the present study. The extraction method developed by Guerin and Sedgley [50] was upgraded with repeated DNA extraction using the optimized CTAB–PVP protocol [48]. The quality and quantity of extracted DNA were sufficient for successful amplification of microsatellite loci. In this study, seven microsatellite loci were used for the identification of the pollen donor and offspring genotypes. The microsatellite primers were chosen based on their amplification efficiency (quality and reproducibility of PCR products) and previously reported genetic parameters [29,30,60], including the number of amplified alleles, the observed heterozygosity, the probability of identity, and the polymorphic information content. All loci were polymorphic and amplified 45 alleles, yielding a minimum of four (EMO3; UDO99–019) and a maximum of eight (ssrOeUA-DCA-3, ssrOeUA-DCA-11, and ssrOeUA-DCA-16) alleles in potential pollen donors, with an average of 6.4 alleles per locus. In both years, pollen donors and offspring populations shared many alleles, with the exception of 14 and 11 unique alleles found in the offspring population in 2017 and 2018, respectively (Table 1). The presence of these unique alleles found in some embryos could be assigned to the fertilization with pollen of cultivars from neighbor olive orchards.

In the pollen donor population, the polymorphic information content (PIC), the exclusion probability (EP), and the probability of identity (PI) were calculated. Considering the polymorphic information content (PIC), all microsatellite loci showed high (>0.500) PIC values that varied from 0.510 for locus EMO3 to 0.766 for locus GAPU101, indicating that these microsatellites were polymorphic and highly informative. The exclusion probability for a single microsatellite locus ranged from 45.8% (EMO3) to 78.3% (GAPU101), and the cumulative exclusion probability was greater than 99.98% and reached using only four microsatellite loci (GAPU101, ssrOeUA-DCA-11, ssrOeUA-DCA-3, and ssrOeUA-DCA-16). These values indicated that the set of microsatellite loci had a very high discriminating capacity for olive varieties, and was supported by a very low combined probability of identity (6 × 10^−9^) and relatively high average PIC value (0.683) (Table 1). PIC, EP, and PI analyses demonstrated that seven microsatellite markers were sufficient to discriminate pollen donor and offspring populations, to exclude almost all unlikely fathers for any given offspring and that the chance of assigning an incorrect offspring genotype to a potential pollen donor was almost null. With the aim to assign the pollen donors to the embryos sampled from ‘Oblica’ trees, the genotyping data of both groups were compared using FaMoz software. The codominant Mendelian inheritance of microsatellite markers has proven to be an efficient system for paternity analysis, as each embryo inherited one allele from the mother tree ‘Oblica’ and another allele at the same locus from the pollen donor cultivar. The embryos were considered as a product of self-fertilization if only maternal alleles were found at all analyzed microsatellite loci. When embryos inherited one of two alleles at each locus from one of the potential pollen donor cultivars, the progeny was considered a product of cross-fertilization (Table 2).

The paternity of 310 (year 2017) and 312 (year 2018) embryos sampled from mother trees was assigned to the most likely pollen donors by calculation of LOD scores (Table 3). Almost all analyzed embryos are assigned to only one possible pollen donor. The simulation for paternity analysis identified a LOD score threshold of 1.50 in 2017 and of 0.86 in 2018. The most likely pollen donor was assigned to 291 (year 2017) and to 303 (year 2018) embryos of all samples (Supplementary Tables S2–S5). An undisputed paternity could not be assigned to 6.0% and to 2.9% of the embryos in 2017 and 2018, respectively. These embryos are denoted as having no likely pollen donor from the set of genotypes planted in the olive orchard. Among the assigned embryos, the male parent genotypes with the highest LOD score above the threshold value for a given parent/offspring pair were determined as the potential pollen donor. The LOD scores were above the estimated threshold for paternity (1.50 and 0.86 for 106 and 252 embryos in 2017 and 2018, respectively). In 2017, only 35% of analyzed embryos were assigned at LOD score above threshold, while for the remaining embryos the LOD scores were lower than the threshold (1.50). These embryos had a larger number of unique alleles (14) and were probably fertilized with a pollen of olive cultivars that were not considered as potential pollen donors in present study. However, the comparison of the most common pollen donors between embryos assigned with only one possible pollen donor (Supplementary Tables S2–S5) and embryos assigned at LOD score above threshold (Table 4) showed exactly the same results in 2017 and 2018, respectively. Based on these findings and in view of the FaMoz program instructions, only embryos assigned at LOD score above threshold were further analyzed (Table 3 and Table 4).

The likely pollen donors assigned to the olive embryos sampled from 11 maternal trees of ‘Oblica’ are shown in Table 3. The cultivars ‘Drobnica’ and ‘Lastovka’ were the major pollen donors for ‘Oblica’ in both years. The percentages of embryos assigned to ‘Drobnica’ were 33.1% and 36.1%, while for ‘Lastovka’ were 16.0% and 47.2%, in 2017 and 2018, respectively. In addition to these two cultivars, a high contribution fathering embryos was found for ‘Istarska bjelica’ (pollen donor in 21.7% of embryos in 2017) and for ‘Dužica’ (pollen donor in 8.7% of embryos in 2018). In addition to these major pollen donors, other cultivars were assigned as possible fathers to 31.1% of embryos in 2017 and to 7.9% of embryos in 2018.

We did not find any embryo having only maternal alleles in any year, highlighting the strong self-incompatibility response of ‘Oblica’. No embryo was a product of cross-pollination with ‘Mastrinka’, a cultivar represented in the orchard by just one tree (Table 5). However, this was not an obstacle for two other cultivars with a single tree in the orchard (‘Buharica’ and ‘Nocellara del Belice’), who fathered 1.2% and 9.4%, and 2.4% and 2.8% of embryos, in 2017 and 2018, respectively.

**Figure 3:**
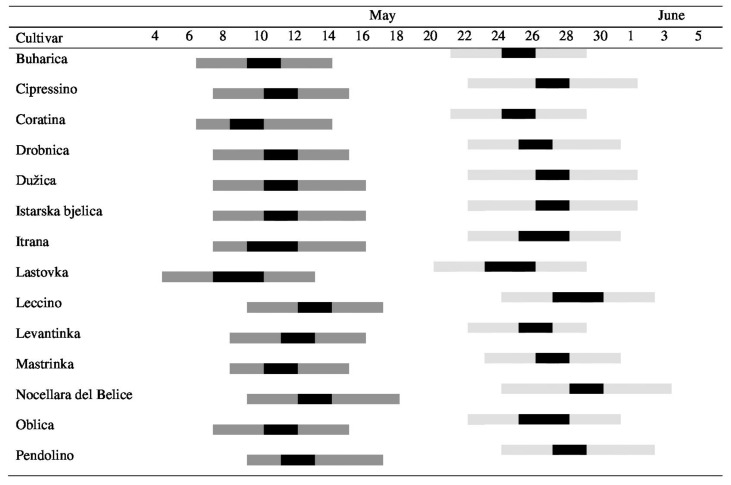
Flowering periods of the olive cultivars during the two years of study: May and June 2017 (light grey) and May 2018 (dark grey). Bars indicate the length of the flowering periods from the beginning (10% flowers open) to the end (the petals darken in color and separate from the calyx in 80% of flowers); full bloom is shown in black.

During the flowering, the daily mean temperatures ranged between 18.4 and 23.6 °C in 2017 and between 15.0 and 24.1 °C in 2018. Bloom periods of the studied cultivars overlapped to some degree in both experimental years (Figure 3). Although the date for the beginning of flowering changed between years, the pattern of flowering was preserved and early blooming cultivars were always the same. ‘Oblica’ mother trees and one of the two most efficient pollen donors according to seed paternity analyses (‘Drobnica’) bloomed simultaneously in both years; however, the flowering period started earlier in ‘Lastovka’, another efficient pollen donor. The constant wind enabled pollen dispersal and pollination among mother trees and pollen donor trees in both seasons (Supplementary Table S6).

Correlation analyses show a weak nonsignificant relationship between the frequency of a given cultivar in the orchard and the proportion of embryo fathered. This was verified in correlation analyses performed including ‘Oblica’ as potential pollen donor (in case of self-fertilization occurs) and without (given the SI previous results confirmed). The correlation coefficients were *r* = −0.0109 (*p* = 0.97) without including ‘Oblica’ and *r* = −0.0094 (*p* = 0.97) with ‘Oblica’ trees included, calculated for both years together. The lack of relationships between these two parameters persists if we take into account the differences in canopy volume between trees (data not shown).

No relationship exists either between the number of embryo fathered by a given pollinizer and the distance to it from the central ‘Oblica’ mother tree (denoted as O4 in Figure 1). The correlation coefficients were *r* = 0.2971 (*p* = 0.32) for the embryos genotyped in 2017, and *r* = 0.1983 (*p* = 0.52) for 2018. The combined effects on the number of embryos fathered in both years confirmed the lack of significant relationship between distance (within the limits here explored) and successful seed paternity (*r* = 0.1072; *p* = 0.73). Interestingly, the proportion of embryo fathered in 2017 and in 2018 were significantly related (*r* = 0.6722; *p* = 0.01), indicating that the success (or lack of) of a given genotype was reproduced in both experimental years.

## 4. Discussion

The knowledge of reproductive biology in olive is crucial for increasing flower fertilization, fruit set, and yield. Most olive cultivars show a high degree of self-incompatibility indicating that interplanting an appropriate number of compatible pollen donor trees is highly advisable. Unfortunately, the information on compatible combinations of olive cultivars is seldom available and there are still doubts on the most reliable methods to test compatibility relationships between cultivars. ‘Oblica’ is the most extensively grown cultivar in Croatia and have a dual purpose, table olives and oil production. Its self- and cross-compatibility with different cultivars was studied observing pollen–pistil interaction in pollination trials [25,46]. In the present study, ‘Oblica’ was the pollen acceptor cultivar and its trees were denoted as the mother trees. During bloom, its flowers were exposed to pollen clouds consisting of pollen from at least fourteen different cultivars, including ‘Oblica’ itself. In this regard, we did not limit the choice of pollen source in any manner. At the harvest period, DNA was extracted directly from uncoated seeds taken from the fruits of mother trees as it was previously done for cultivars grown in Australia [28] and Italy [31].

The effectiveness of SSR markers has been demonstrated in olive for cross-compatibility validation and for seed paternity assignment. Several authors have used a different number of microsatellite markers, ranging from two [31], four [27,29,45], seven [30], or eight [28,44], to ten [22]. In our study, the use of seven microsatellite primers was sufficient to determine seed paternity in ‘Oblica’ fruit as predicted by the high polymorphic information content (0.683), the high exclusion probability (0.9998), and the low probability of identity (6 × 10^−9^) [55,56] (Table 1). Using these markers, almost all analyzed embryos were successfully assigned to only one possible pollen donor. These results confirmed that a relatively low number of SSR markers is sufficient for paternal assignment with a high degree of confidence. As shown for other olive cultivars [28,30,44], high levels of codominant polymorphism, characteristic for SSR loci, positively influence the combined parentage exclusion probabilities (EP > 0.996) and give rise to highly accurate assignments of the pollen donors [61,62].

Our results clearly show that fertilization was in all cases the result of cross-pollination (Table 4). This situation confirms the strong self-incompatibility response of ‘Oblica’. The occurrence of self-fertilization was not detected either in 1440 embryos extracted from different cultivars in a recent study carried out under open pollination [22]. The low occurrence of self-fertilization is not unusual in olive, but emphasizes the importance of pollination designs with suitable pollinizers, especially in isolated orchards and new areas of olive growing. Secondly, the present study shows that cultivars ‘Drobnica’ and ‘Lastovka’ were the most efficient pollen donors for ‘Oblica’. In addition to these two cultivars, ‘Istarska bjelica’ and ‘Dužica’ fathered an important proportion of embryos (Table 4). The success of these cultivars in fathering ‘Oblica’ embryos was confirmed in 2017 and 2018, suggesting the existence of intrinsic characteristics of those cultivars that make them good pollinizers for ‘Oblica’. All this information is of great significance to confirm ‘Oblica’ dependence from cross-pollination and the compatibility relationships with Croatian cultivars that can be chosen as suitable pollinizers.

Up to the present, we have used different methods in the assessment of compatibility relationships within and among the most important Croatian cultivars (‘Drobnica’, ‘Lastovka’, ‘Levantinka’, and ‘Oblica’) and the most common Italian cultivars in newly planted orchards (‘Leccino’ and ‘Pendolino’). Pollen–pistil interaction parameters leading to fruit set (pollen germination on the stigma, pollen tube growth in the style, and fertilization) reported in an earlier study [25] showed different compatibility relationships between ‘Oblica’ and potential pollen donor cultivars (Table 6). In those experiments, only the pollen of different single donors was directly applied to the stigmas of ‘Oblica’, and pollen germination and pollen tube growth in the style were considered as indicators of compatibility. The relationships between cultivars based on pollen–pistil interaction and fruit set data [25,46] are consistent with those obtained by paternity analyses in the present study (Table 6). In this regard, a compatible relationship was established for ‘Oblica’ with ‘Levantinka’ and ‘Pendolino’. Paternity analyses confirmed such compatibility relationship since paternity was confirmed in six (‘Levantinka’) and five (‘Pendolino’) embryos. Compared to ‘Levantinka’ and ‘Pendolino’, we did not observe clear compatibility response in ‘Oblica’ when ‘Lastovka’ was used as pollen donor. However, this cultivar was among the major pollen donors in the paternity analyses with 38% embryos fathered in two years. Pollen–pistil interaction underlined self-incompatibility response in ‘Oblica’, showing a clear preference for cross-pollen, a situation confirmed by the paternity analyses of this study. Once confirmed, the self-incompatibility of ‘Oblica’ and the differential acceptance of cross-pollen depending on the donor, a selection of the most suitable pollinizers must follow. Global warming may increase ‘Oblica’ dependence from more rapid pollen tube growth provided by cross-pollen since it may reduce ovule longevity and the duration of stigma receptivity. Nonetheless, when selecting pollen donor genotypes, the chosen pollinizer not only has to be compatible with the main cultivar, but also must accomplish some prerequisites. The most important ones are presenting sufficient bloom overlap with the main variety, having a regular bearing (not often alternating), and preferably having the same industrial purpose (table olives or oil production) for facilitating the commercialization of the product [11,28].

The importance of simultaneous flowering with mother trees for successful fertilization can be discussed here. Large overlap in blooming has to be selected as one of the main characteristics for a suitable pollinizer. Poor overlap in flowering is enough to understand the absence of particular parental alleles in the progenies, although a cross-compatibility relation may exist between these two cultivars. Therefore, the phenology of bloom has to be followed in all experimental sites during several years to select the best pollinizer for the main variety. In our study, the most efficient pollen donors bloomed almost simultaneously with ‘Oblica’ in both years. However, the simultaneous flowering of ‘Cipressino’ and the high number of trees present in the orchard (15.9%) was not a sufficient guarantee of success since only 1.6% of embryos in 2018 (zero in 2017) were a product of cross-fertilization by ‘Cipressino’ pollen.

Pollination success can be also strongly affected by the abundance and proximity of the pollen source. In this regard, a low percentage of embryos fathered by a given pollen donor does not preclude its compatibility with the main cultivar, only its secondary role in comparison to another, perhaps over-represented pollen donor. This situation was noted for ‘Mastrinka’, a cultivar presented in the orchard with just one tree. However, two other cultivars, ‘Buharica’ and ‘Nocellara del Belice’, achieved a higher success under the same conditions. The spatial relationship between the mother trees, the pollen donors, and the frequencies of pollen donors are represented in Figure 1 and Table 4 and Table 5. Pollen donors at the shortest distance and with the highest number of trees in the orchard were expected to be among the most successful. Strikingly, the dominant pollinizers in this study were not the most frequent or the closest neighbors to the maternal trees of ‘Oblica’, confirming that bloom phenology and cross-compatibility have a key role in pollinizer success. We have to mention, however, that all genotypes scrutinized were within or close to the most effective pollination distance of 30–40 m for maximizing olive yield [10,63] (Table 5). In addition, the abundance of pollen produced in the pollen donor cultivar can change significantly between off and on years, depending on the low or high level of flowering, respectively. This situation can change the frequency of pollinizer in the mother tree.

Potential pollen donor cultivars not fathering embryos suggests cross-incompatibility with the pollen acceptor cultivars if blooming coincides in time. On the contrary, correlation analyses show a nonsignificant relationship between the frequency of a given cultivar in the orchard and the proportion of embryo fathered (see Results). In accordance to our research, Mookerjee et al. [28] found a weak correlation between the number of trees of any given genotype in an olive grove and the number of fathered embryos. Pollen donors for 6.0% (2017) and 2.9% (2018) of embryos in our study could not be identified. This finding suggests that mother trees were pollinated by unidentified genotypes from the olive orchard or from trees outside the orchard. Similar results were observed previously [22,28,30], indicating that certain amounts of olive pollen is carried by wind from larger distances contributing to fruit set when intended pollen donor trees fail to fertilize the flowers [64].

Lack of overlap in blooming, low frequency in the orchard, and/or long distance to mother trees can partially explain a low proportion of embryo fathered by a potential pollen donor. Nonetheless, pollen–pistil interaction analyses should always be performed to confirm incompatibility relationships between cultivars. Observations of pollen–pistil interaction have been employed to study compatibility relationships in many crops [25,65,66,67,68,69,70,71]. Studies of pollen germination on the stigma, pollen tube growth in the style, and fertilization success were extensively used to determine the compatibility relationships between olive cultivars [25]. In those studies, self-incompatibility reaction was characterized by limited pollen tube growth in the style of the recipient flowers. On the contrary, pollen germination after compatible cross-pollination is abundant and massive pollen tube competition occurred in the stigma and style, leading to successful fertilization. Abundant and rapid pollen tube growth and fertilization success highly correlates with high fruit set [23]. In any case, pollen–pistil interaction studies are compatible with paternity analyses, despite some discrepancies that may arise. In paternity studies, cultivar ‘Lastovka’ is underlined as one of the most efficient pollen donors for ‘Oblica’. However, earlier studies gave the prevalence to other pollen donors [25,46]. Higher number of trees, their proximity, and abundant pollen production probably gave preference to this variety in our paternity analyses.

Reproductive analyses usually encompass all processes of the progamic phase (pollen–pistil interaction) and post fertilization processes (fruit set and early fruit development). These analyses have the advantages to show early signs of inter-incompatibility between olive cultivars. On the contrary, in paternity analysis, seeds from the fruits at the final stages of reproductive development or developing seedlings are used. Although late measured, seed paternity represents an unquestionable fitness success for the pollen donor genotype. Seed paternity analyses by microsatellite markers have been proven effective tools here and previously [28] in the assessment of compatibility between olive cultivars when potential pollen donors are multiple and pollen is not directly applied by hand to the recipient flowers.

## 5. Conclusions

The knowledge of self- and cross-compatibility relationships is important for olive breeders and for growers when establishing pollination designs in their orchards. Our results confirm the preferential allogamy in cultivar ‘Oblica’ and the success of pollinizers that were not always the most represented or closest to the maternal trees. Large bloom overlap and cross-compatibility relationships seem to be the major drivers in seed paternity success. The information on the most likely pollen donors for cultivar ‘Oblica’ obtained in this study can be used for growers when selecting suitable pollinizers for olive orchard designs.

## Figures and Tables

**Table 1:** Number of alleles (*n*), polymorphic information content (PIC), probability of identity (PI), and exclusion probability (EP) for potential pollen donors calculated using seven microsatellite loci, and offspring specific alleles detected in 2017 and 2018.

Microsatellite Locus	*n*	PIC	PI	EP	Offspring Specific Alleles	
					2017	2018
ssrOeUA-DCA–3	8	0.726	0.0509	0.7438	2	/
ssrOeUA-DCA–9	6	0.690	0.0638	0.7040	3	3
ssrOeUA-DCA–11	8	0.733	0.0424	0.7579	3	3
ssrOeUA-DCA–16	8	0.707	0.0577	0.7252	1	2
GAPU101	7	0.766	0.0376	0.7830	1	/
EMO3	4	0.510	0.2142	0.4577	4	3
UDO99–019	4	0.652	0.0973	0.6285	/	/
Total	45	0.683 *	6 × 10^−9^ **	0.9998 ***	14	11

* Average of PIC values for 7 SSR loci; ** product of PI values for 7 SSR loci; *** cumulative value over all 7 SSR loci.

**Table 2:** The fundamentals of paternity analysis using SSR markers. The numbers are the allele sizes in bp, amplified in mother tree ‘Oblica’, different embryos, and potential pollen donors. The genotype profile of mother tree ‘Oblica’ includes two alleles of 192 and 218 bp. All the presented embryos are the result of cross-fertilization; each embryo inherited one allele from mother tree ‘Oblica’, 192 bp (E1 and E2), or 218 bp (E3 and E4), and the other allele from corresponding pollen donor cultivar ‘Drobnica’ (E1), ‘Leccino’ (E2), ‘Lastovka’ (E3), and ‘Istarska bjelica’ (E4).

Pollen Donor and Alleles Size		Mother Tree ‘Oblica’	Embryo Identity	Embryo Genotype
‘Drobnica’	194	192	E1	192
	207			207
‘Leccino’	199		E2	192
	201			201
‘Lastovka’	192	218	E3	218
	194			194
‘Istarska bjelica’	199		E4	218
	201			201

**Table 3:** Number of embryos unassigned, assigned with only one possible pollen donor, and assigned at LOD score above threshold (LOD score above 1.5 in 2017 and 0.86 in 2018).

Embryo Assignment	Number of Embryos	
	2017	2018
Total	310	312
No likely pollen donor	19	9
Assigned with only one possible pollen donor	291	303
Assigned at LOD score above threshold	106	252

**Table 4:** Number of embryos from ‘Oblica’ mother trees (O1–O6) assigned to each potential pollen donor in 2017 and 2018.

Pollen Donor	Mother Tree												
	2017							2018					
	O1	O2/1	O3	O4	O5	O6	Total	O1	O2/2	O3	O5	O6	Total
‘Buharica’	3	0	1	2	4	0	10	0	0	1	2	0	3
‘Cipressino’	0	0	0	0	0	0	0	2	2	0	0	0	4
‘Coratina’	0	0	0	1	1	0	2	0	0	0	0	0	0
‘Drobnica’	7	3	9	4	6	4	33	7	5	49	28	2	91
‘Dužica’	0	1	1	0	0	1	3	5	7	5	4	1	22
‘Istarska bjelica’	1	2	4	8	3	5	23	2	0	1	0	0	3
‘Itrana’	1	1	1	1	1	0	5	0	0	0	1	0	1
‘Lastovka’	2	1	1	4	5	4	17	28	23	14	15	39	119
‘Leccino’	0	0	1	0	1	0	2	0	0	0	0	0	0
‘Levantinka’	1	1	0	2	1	0	5	0	1	0	0	0	1
‘Mastrinka’	0	0	0	0	0	0	0	0	0	0	0	0	0
‘Nocellara del Belice’	1	0	1	0	1	0	3	3	0	3	0	0	6
‘Oblica’	0	0	0	0	0	0	0	0	0	0	0	0	0
‘Pendolino’	1	0	1	0	1	0	3	0	0	2	0	0	2
Total	17	9	20	22	24	14	106	47	38	75	50	42	252

**Table 5:** List of potential pollen donor cultivars, associated percentages of the trees of each pollen donor in the orchard (number of pollen donor trees/total number of trees in the orchard x100), closest distance of pollen donor tree to O4 ‘Oblica’ mother tree, and percentage of assigned embryos (%) in the two experimental years.

Pollen Donor Cultivar	Percentage of Trees (%)	Closest Distance to ‘Oblica’ Mother Tree (m)	Percentage of Embryos (%)
‘Buharica’	0.8	43	3.6
‘Cipressino’	15.5	10	1.1
‘Coratina’	1.6	9	0.6
‘Drobnica’	5.4	18	34.6
‘Dužica’	1.6	20	7.0
‘Istarska bjelica’	23.2	21	7.3
‘Itrana’	2.3	6	1.7
‘Lastovka’	7.7	25	38.0
‘Leccino’	8.5	6	0.6
‘Levantinka’	2.3	23	1.7
‘Mastrinka’	0.8	9	0
‘Nocellara del Belice’	0.8	32	2.5
‘Oblica’	27.1	-	0
‘Pendolino’	2.3	10	1.4

**Table 6:** An overview of compatibility relationship in ‘Oblica’ using different methods.

Pollen Donor Cultivar	Pollen Germination ^1^	Pollen Tube Growth ^1^	Fertilization Success ^1^	Final Fruit Set ^2^	Seed Paternity
‘Lastovka’	Germination fairly supported	PTG fairly supported	Low	Cross-compatible	Highly cross-compatible
‘Leccino’	Germination supported	PTG supported	Medium	Cross-compatible	Cross-compatible
‘Levantinka’	Germination abundantly supported	PTG abundantly supported	High	Cross-compatible	Cross-compatible
‘Oblica’	Germination fairly supported	PTG fairly supported	Low	Partially SI	Self-incompatible
‘Pendolino’	Germination abundantly supported	PTG abundantly supported	High	Cross-compatible	Cross-compatible

^1^ Ref. [25]; ^2^ Ref. [46].

## Data Availability

The original contributions generated for this study are included in the article/Supplementary Material; further inquiries can be directed to the corresponding author.

## Supplementary Materials

The following are available online at https://www.mdpi.com/article/10.3390/plants10112356/s1, Table S1. List of SSR primers and their sequences; Table S2. List of embryos, their pollen donors and LOD score values in 2017; Table S3. Number of embryos from ‘Oblica’ variety assigned to each potential pollen donor in 2017; Table S4. List of embryos, their pollen donors and LOD score values in 2018; Table S5. Number of embryos from ‘Oblica’ variety assigned to each potential pollen donor in 2018; Table S6. Wind direction (east, E; north, N; northeast, NE; northwest, NW; south, S; southeast, SE; southwest, SW; west, W) and strength (2–5 m/s, light, 1; 5–9.9 m/s, moderate, 2; >9.9 m/s, strong, 3) during the flowering period in 2017 (light grey) and 2018 (dark grey).
