# Supplementary material for: Seed Paternity Analysis Using SSR Markers to Assess Successful Pollen Donors in Mixed Olive Orchards
Source: Plants (Basel). 2021 Oct 31;10(11):2356. doi: 10.3390/plants10112356 (PMC8624852; doi:10.3390/plants10112356)
Supplement: Supplementary file 1 [file plants-10-02356-s001.zip › plants-1413159-supplementary.pdf]

Supplementary Material

Table S1. List of SSR primers and their sequences.

No.	Locus	Primer sequence (5'-3')	Repeat motif
1	ssrOeUA-DCA-3	F: *CCCAAGCGGAGGTGTATATTGTTAC<br>R: TGCTTTTGTCGTGTTTGAGATGTTG	$(GA)_{19}$
2	ssrOeUA-DCA-9	F: *AATCAAAGTCTTCCTTCTCATTTCG<br>R: GATCCTTCCAAAAGTATAACCTCTC	$(Ga)_{23}$
3	ssrOeUA-DCA-11	F: *GATCAAACTACTGCACGAGAGAG<br>R: TGTCTCAGTGAACCCTTAAACC	$(GA)_{26}(GGGA)_4$
4	ssrOeUA-DCA-16	F: *TTAGGTGGGATTCTGTAGATGGTTG<br>R: TTTTAGGTGAGTTCATAGAATTAGC	$(GT)_{13}(GA)_{29}$
5	GAPU101	F: *CATGAAAGGAGGGGGACATA<br>R: GGCACTTGTTGTGCAGATTG	$(GA)_8(G)_3(AG)_3$
6	EMO3	F: *GGTGTAGCCCAAGCCCTTAT<br>R: GCATGACCGTGGTGTAAGT	$(CA)_7$
7	UDO99-019	F: *CCCTTGTAGCCTCGTCTTG<br>R: GGCCTGATCATCGATACCTC	$(GT)_{20}(AT)_5$

* - primers elongated for M13 (-21) 18 bp sequence (5’-TGTAAAACGACGGCCAGT-3’) at their 5’ ends

Table S2. List of embryos, their pollen donors and LOD score values in 2017.

Embryo	Pollen donor	LOD score	Embryo	Pollen donor	LOD score
293	'Pendolino'	3.39	146	'Istarska bjelica'	1.88
275	'Pendolino'	2.88	223	'Istarska bjelica'	1.88
256	'Istarska bjelica'	2.61	302	'Istarska bjelica'	1.88
169	'Buharica'	2.59	306	'Istarska bjelica'	1.88
201	'Drobnica'	2.50	137	'Istarska bjelica'	1.86
10	'Drobnica'	2.41	162	'Buharica'	1.86
140	'Drobnica'	2.41	207	'Dužica'	1.85
276	'Drobnica'	2.41	40	'Lastovka'	1.84
80	'Istarska bjelica'	2.40	30	'Lastovka'	1.83
218	'Drobnica'	2.38	114	'Istarska bjelica'	1.77
29	'Istarska bjelica'	2.31	115	'Istarska bjelica'	1.77
295	'Drobnica'	2.28	55	'Leccino'	1.77
287	'Levantinka'	2.10	113	'Lastovka'	1.77
261	'Istarska bjelica'	2.08	166	'Lastovka'	1.77
310	'Istarska bjelica'	2.08	278	'Drobnica'	1.76
270	'Drobnica'	2.07	308	'Dužica'	1.75
61	'Buharica'	2.07	15	'Istarskabjelica'	1.73
219	'Buharica'	2.07	107	'Istarska bjelica'	1.73
109	'Levantinka'	2.02	121	'Istarska bjelica'	1.73
158	'Levantinka'	2.02	144	'Istarska bjelica'	1.73
173	'Itrana'	2.01	23	'Drobnica'	1.72
56	'Levantinka'	2.01	63	'Drobnica'	1.72
132	'Levantinka'	2.01	66	'Drobnica'	1.72
34	'Coratina'	1.98	67	'Drobnica'	1.72
11	'Drobnica'	1.97	226	'Drobnica'	1.72
163	'Drobnica'	1.97	227	'Drobnica'	1.72
232	'Drobnica'	1.97	309	'Lastovka'	1.70
92	'Coratina'	1.96	123	'Istarska bjelica'	1.66
160	'Drobnica'	1.94	156	'Istarska bjelica'	1.66
184	'Drobnica'	1.94	208	'Istarska bjelica'	1.66
213	'Itrana'	1.93	228	'Istarska bjelica'	1.66
7	'Buharica'	1.93	288	'Istarska bjelica'	1.66
59	'Lastovka'	1.93	297	'Istarska bjelica'	1.66
152	'Drobnica'	1.89	141	'Lastovka'	1.66
165	'Drobnica'	1.89	38	'Lastovka'	1.65
286	'Drobnica'	1.89	54	'Lastovka'	1.65
290	'Drobnica'	1.89	182	'Lastovka'	1.65
221	'Buharica'	1.89	233	'Dužica'	1.64
89	'Istarska bjelica'	1.88	42	'Lastovka'	1.64

Embryo	Pollen donor	LOD score
214	’Drobnica’	1.62
72	’Leccino’	1.56
168	’Pendolino’	1.55
124	’Lastovka’	1.55
210	’Lastovka’	1.55
175	’Drobnica’	1.53
304	’Drobnica’	1.53
264	’Nocellara’	1.53
122	’Itrana’	1.53
294	’Itrana’	1.53
133	’Buharica’	1.53
170	’Buharica’	1.53
241	’Buharica’	1.53
279	’Buharica’	1.53
13	’Nocellara’	1.51
25	’Nocellara’	1.51
24	’Lastovka’	1.51
130	’Lastovka’	1.51
149	’Lastovka’	1.51
260	’Lastovka’	1.51
78	’Itrana’	1.50
27	’Drobnica’	1.50
119	’Drobnica’	1.50
197	’Drobnica’	1.50
206	’Drobnica’	1.50
244	’Drobnica’	1.50
248	’Drobnica’	1.50
303	’Drobnica’	1.50
289	’Drobnica’	1.42
104	’Itrana’	1.41
301	’Istarska bjelica’	1.41
37	’Buharica’	1.41
99	’Buharica’	1.41
155	’Dužica’	1.41
191	’Dužica’	1.41
76	’Istarska bjelica’	1.40
127	’Istarska bjelica’	1.40
73	’Istarska bjelica’	1.36
135	’Istarska bjelica’	1.36

Embryo	Pollen donor	LOD score
200	’Buharica’	1.36
224	’Buharica’	1.36
83	’Leccino’	1.35
68	’Itrana’	1.33
193	’Itrana’	1.33
139	’Buharica’	1.33
159	’Buharica’	1.33
202	’Buharica’	1.33
242	’Lastovka’	1.33
273	’Lastovka’	1.33
62	’Drobnica’	1.32
81	’Dužica’	1.31
26	Cempresino	1.29
85	Cempresino	1.29
179	Cempresino	1.29
296	’Levantinka’	1.29
70	’Buharica’	1.27
171	’Buharica’	1.27
180	’Buharica’	1.27
183	’Buharica’	1.27
217	’Buharica’	1.27
234	’Buharica’	1.27
268	’Buharica’	1.27
22	’Itrana’	1.26
249	’Itrana’	1.26
307	’Istarska bjelica’	1.26
1	’Buharica’	1.26
20	’Buharica’	1.26
185	’Istarska bjelica’	1.23
138	’Dužica’	1.19
178	’Dužica’	1.19
215	’Lastovka’	1.17
161	’Istarska bjelica’	1.16
298	’Istarska bjelica’	1.14
253	’Drobnica’	1.13
19	’Drobnica’	1.12
84	’Dužica’	1.11
2	’Drobnica’	1.10
43	’Drobnica’	1.10

Embryo	Pollen donor	LOD score	Embryo	Pollen donor	LOD score
46	'Drobnica'	1.10	49	'Istarska bjelica'	0.83
50	'Drobnica'	1.10	71	'Istarska bjelica'	0.83
239	'Drobnica'	1.10	103	'Istarska bjelica'	0.83
269	'Drobnica'	1.10	267	'Istarska bjelica'	0.83
5	'Lastovka'	1.10	299	'Istarska bjelica'	0.83
136	'Lastovka'	1.10	157	'Itrana'	0.81
225	'Lastovka'	1.10	44	'Lastovka'	0.79
211	'Dužica'	1.08	82	'Lastovka'	0.79
236	'Dužica'	1.08	91	'Lastovka'	0.79
220	'Istarska bjelica'	1.06	128	'Lastovka'	0.79
145	'Istarska bjelica'	1.05	188	'Coratina'	0.78
258	'Istarska bjelica'	1.05	240	'Drobnica'	0.77
88	'Lastovka'	1.03	259	'Drobnica'	0.77
125	'Lastovka'	1.03	305	'Drobnica'	0.77
86	'Dužica'	1.01	8	'Lastovka'	0.76
177	'Dužica'	1.01	102	'Lastovka'	0.76
251	'Dužica'	1.01	118	'Lastovka'	0.76
105	'Drobnica'	1.00	194	'Lastovka'	0.76
271	'Lastovka'	0.99	204	'Lastovka'	0.76
36	'Drobnica'	0.97	230	'Itrana'	0.75
79	'Drobnica'	0.97	281	'Itrana'	0.75
129	'Drobnica'	0.97	196	StaraMaslina	0.75
167	'Drobnica'	0.97	45	'Buharica'	0.75
283	'Drobnica'	0.97	57	'Buharica'	0.75
277	'Lastovka'	0.97	142	'Buharica'	0.75
112	'Buharica'	0.96	254	'Buharica'	0.75
75	'Lastovka'	0.92	21	'Leccino'	0.74
98	'Lastovka'	0.92	87	'Lastovka'	0.74
116	'Lastovka'	0.92	90	'Istarska bjelica'	0.73
126	'Lastovka'	0.92	148	'Istarska bjelica'	0.73
190	'Lastovka'	0.92	154	'Istarska bjelica'	0.73
17	'Drobnica'	0.91	285	'Istarska bjelica'	0.73
255	'Dužica'	0.90	203	'Istarska bjelica'	0.67
51	'Lastovka'	0.90	33	'Dužica'	0.66
117	'Pendolino'	0.89	265	'Lastovka'	0.65
111	'Istarska bjelica'	0.88	32	'Buharica'	0.63
187	'Istarska bjelica'	0.84	58	'Lastovka'	0.63
181	'Buharica'	0.84	31	'Istarska bjelica'	0.61
39	'Istarska bjelica'	0.83	53	'Istarska bjelica'	0.61

Embryo	Pollen donor	LOD score
60	’Istarska bjelica’	0.61
69	’Istarska bjelica’	0.61
134	’Istarska bjelica’	0.61
198	’Istarska bjelica’	0.61
250	’Istarska bjelica’	0.61
252	’Istarska bjelica’	0.61
212	’Nocellara’	0.61
280	’Drobnica’	0.60
131	’Istarska bjelica’	0.58
65	’Dužica’	0.56
245	’Dužica’	0.56
16	’Lastovka’	0.56
195	’Lastovka’	0.56
209	’Lastovka’	0.56
3	’Lastovka’	0.54
6	’Lastovka’	0.54
243	’Itrana’	0.52
262	’Drobnica’	0.47
300	’Lastovka’	0.45
110	’Nocellara’	0.44
247	’Dužica’	0.37
47	’Lastovka’	0.37
48	’Lastovka’	0.37
93	’Lastovka’	0.37
96	’Lastovka’	0.37
108	’Lastovka’	0.37
222	’Lastovka’	0.37
28	’Istarska bjelica’	0.35
35	’Istarska bjelica’	0.35
64	’Istarska bjelica’	0.35
77	’Istarska bjelica’	0.35
94	’Istarska bjelica’	0.35
150	’Istarska bjelica’	0.35
153	’Istarska bjelica’	0.35
164	’Istarska bjelica’	0.35
205	’Istarska bjelica’	0.35
229	’Istarska bjelica’	0.35
246	’Istarska bjelica’	0.35
282	’Istarska bjelica’	0.35

Embryo	Pollen donor	LOD score
284	’Istarska bjelica’	0.35
292	’Istarska bjelica’	0.35
74	’Drobnica’	0.25
151	’Drobnica’	0.25
235	’Drobnica’	0.25
263	’Drobnica’	0.25
266	’Drobnica’	0.25
147	’Nocellara’	0.22
12	’Lastovka’	0.22
257	’Lastovka’	0.21
52	’Lastovka’	0.15
97	’Lastovka’	0.10
231	’Lastovka’	0.10
274	’Lastovka’	0.10
100	’Lastovka’	0.03
9	’Lastovka’	0.02
174	’Lastovka’	0.02
101	’Istarska bjelica’	0.01
4	No likely pollen donor	
14	No likely pollen donor	
18	No likely pollen donor	
41	No likely pollen donor	
95	No likely pollen donor	
106	No likely pollen donor	
120	No likely pollen donor	
143	No likely pollen donor	
172	No likely pollen donor	
176	No likely pollen donor	
186	No likely pollen donor	
189	No likely pollen donor	
192	No likely pollen donor	
199	No likely pollen donor	
216	No likely pollen donor	
237	No likely pollen donor	
238	No likely pollen donor	
272	No likely pollen donor	
291	No likely pollen donor	

Table S3. Number of embryos from 'Oblica' variety assigned to each potential pollen donor in 2017.

	Embryos assigned with only one possible pollen donor	Embryos assigned at LOD score above threshold (1,5)
'Buharica'	33	10
'Cipresino'	3	0
'Coratina'	3	2
**'Drobnica'**	**60**	**33**
'Dužica'	19	3
**'Istarska bjelica'**	**72**	**23**
'Itrana'	14	5
**'Lastovka'**	**66**	**17**
'Leccino'	4	2
'Levantinka'	6	5
'Mastrinka'	1	0
'Nocellara'	6	3
'Oblica'	0	0
'Pendolino'	4	3
Sum	291	106

Table S4. List of embryos, their pollen donors and LOD score values in 2018.

Embryo	Pollen donor	LOD score	Embryo	Pollen donor	LOD score
125	'Drobnica'	4.79	64	'Drobnica'	2.81
1	'Drobnica'	4.00	104	'Drobnica'	2.81
75	'Drobnica'	4.00	58	'Lastovka'	2.77
98	'Drobnica'	4.00	269	'Lastovka'	2.71
73	'Drobnica'	3.99	185	'Lastovka'	2.70
92	'Drobnica'	3.81	235	'Lastovka'	2.69
39	'Drobnica'	3.60	121	'Drobnica'	2.65
47	'Drobnica'	3.60	229	'Lastovka'	2.65
56	'Drobnica'	3.60	251	'Lastovka'	2.65
90	'Drobnica'	3.59	270	'Lastovka'	2.65
106	'Drobnica'	3.55	22	'Drobnica'	2.62
124	'Drobnica'	3.54	128	'Lastovka'	2.61
69	'Drobnica'	3.53	70	'Drobnica'	2.56
120	'Drobnica'	3.52	117	'Drobnica'	2.56
129	'Drobnica'	3.52	122	'Drobnica'	2.56
108	'Lastovka'	3.38	4	'Drobnica'	2.55
135	'Drobnica'	3.31	89	'Drobnica'	2.55
206	'Lastovka'	3.28	74	'Drobnica'	2.52
250	'Lastovka'	3.28	214	'Drobnica'	2.52
259	'Lastovka'	3.28	255	'Drobnica'	2.52
97	'Lastovka'	3.17	294	'Lastovka'	2.51
181	'Lastovka'	3.10	137	'Drobnica'	2.50
236	'Lastovka'	3.08	30	'Lastovka'	2.45
49	'Drobnica'	3.03	133	'Lastovka'	2.44
114	'Drobnica'	3.02	247	'Lastovka'	2.44
219	'Lastovka'	2.99	248	'Lastovka'	2.44
222	'Lastovka'	2.99	249	'Lastovka'	2.44
227	'Lastovka'	2.99	273	'Lastovka'	2.44
234	'Lastovka'	2.99	220	'Lastovka'	2.42
223	'Lastovka'	2.97	266	'Lastovka'	2.42
126	'Lastovka'	2.84	267	'Lastovka'	2.42
132	'Lastovka'	2.83	274	'Lastovka'	2.42
63	'Drobnica'	2.82	113	'Drobnica'	2.37
79	'Drobnica'	2.82	241	'Lastovka'	2.37
84	'Drobnica'	2.82	8	'Drobnica'	2.35
107	'Drobnica'	2.82	11	'Drobnica'	2.35
291	'Lastovka'	2.82	102	'Drobnica'	2.35
9	'Drobnica'	2.81	66	'Drobnica'	2.34
43	'Drobnica'	2.81	50	'Drobnica'	2.31
174	'Lastovka'	2.30	172	'Lastovka'	2.27

Embryo	Pollen donor	LOD score	Embryo	Pollen donor	LOD score
224	'Lastovka'	2.25	277	'Cempresino'	1.85
237	'Lastovka'	2.25	26	'Drobnica'	1.84
271	'Lastovka'	2.25	61	'Drobnica'	1.84
282	'Lastovka'	2.25	93	'Drobnica'	1.84
287	'Lastovka'	2.25	115	'Drobnica'	1.84
3	'Lastovka'	2.23	154	'Lastovka'	1.84
190	'Lastovka'	2.23	5	'Drobnica'	1.83
62	'Lastovka'	2.22	14	'Drobnica'	1.83
145	'Lastovka'	2.22	15	'Drobnica'	1.83
161	'Lastovka'	2.18	40	'Drobnica'	1.83
167	'Lastovka'	2.16	44	'Lastovka'	1.83
187	'Lastovka'	2.16	53	'Drobnica'	1.83
209	'Drobnica'	2.15	143	'Lastovka'	1.83
65	'Drobnica'	2.12	146	'Lastovka'	1.83
131	'Drobnica'	2.12	157	'Lastovka'	1.83
57	'Dužica'	2.10	160	'Lastovka'	1.83
127	'Drobnica'	2.10	162	'Lastovka'	1.83
130	'Dužica'	2.10	195	'Lastovka'	1.83
34	'Lastovka'	2.09	272	'Lastovka'	1.79
111	'Dužica'	2.09	103	'Lastovka'	1.74
76	'Lastovka'	2.07	101	'Lastovka'	1.73
86	'Lastovka'	2.07	149	'Lastovka'	1.73
139	'Drobnica'	2.06	217	'Lastovka'	1.72
211	'Drobnica'	2.06	297	'Lastovka'	1.72
213	'Drobnica'	2.06	118	'Dužica'	1.70
304	'Drobnica'	2.06	202	'Lastovka'	1.70
37	'Drobnica'	2.03	12	'Lastovka'	1.69
141	'Drobnica'	2.02	177	'Lastovka'	1.69
88	'Nocellara'	1.97	2	'Lastovka'	1.67
152	'Lastovka'	1.95	29	'Istarska bjelica'	1.67
186	'Lastovka'	1.95	281	'Drobnica'	1.67
59	'Lastovka'	1.92	303	'Drobnica'	1.66
184	'Lastovka'	1.92	260	'Drobnica'	1.65
18	'Lastovka'	1.90	91	'Buharica'	1.61
119	'Lastovka'	1.90	95	'Buharica'	1.61
179	'Lastovka'	1.90	96	'Itrana'	1.61
207	'Lastovka'	1.87	138	'Buharica'	1.61
136	'Drobnica'	1.86	178	'Lastovka'	1.58
196	'Lastovka'	1.86	180	'Lastovka'	1.58
197	'Lastovka'	1.86	290	'Lastovka'	1.57
263	'Lastovka'	1.85	292	'Lastovka'	1.57

Embryo	Pollen donor	LOD score
32	’Dužica’	1.56
35	’Dužica’	1.56
100	’Dužica’	1.56
33	’Dužica’	1.55
38	’Drobnica’	1.55
85	’Dužica’	1.55
243	’Lastovka’	1.55
194	’Lastovka’	1.53
203	’Lastovka’	1.53
150	’Lastovka’	1.46
176	’Lastovka’	1.46
41	’Drobnica’	1.45
80	’Lastovka’	1.45
42	’Drobnica’	1.44
68	’Pendolino’	1.42
309	’Lastovka’	1.42
256	’Cempresino’	1.41
252	’Lastovka’	1.40
200	’Lastovka’	1.39
232	’Drobnica’	1.39
45	’Drobnica’	1.38
228	’Cempresino’	1.38
293	’Cempresino’	1.38
110	’Drobnica’	1.37
144	’Lastovka’	1.37
296	’Lastovka’	1.35
21	’Drobnica’	1.34
25	’Pendolino’	1.34
233	’Nocellara’	1.33
36	’Drobnica’	1.31
46	’Drobnica’	1.31
239	’Lastovka’	1.31
7	’Lastovka’	1.30
109	’Lastovka’	1.30
165	’Lastovka’	1.29
201	’Lastovka’	1.29
208	’Lastovka’	1.29
204	’Lastovka’	1.28
231	’Nocellara’	1.28
265	’Lastovka’	1.27
205	’Lastovka’	1.26

Embryo	Pollen donor	LOD score
284	’Levantinka’	1.25
240	’Istarska bjelica’	1.24
253	’Lastovka’	1.24
262	’Istarska bjelica’	1.24
278	’Lastovka’	1.23
283	’Dužica’	1.23
295	’Lastovka’	1.23
210	’Dužica’	1.19
215	’Dužica’	1.19
216	’Dužica’	1.19
238	’Dužica’	1.19
245	’Dužica’	1.19
298	’Dužica’	1.19
300	’Dužica’	1.19
305	’Dužica’	1.19
311	’Dužica’	1.19
276	’Dužica’	1.18
302	’Dužica’	1.18
307	’Dužica’	1.17
153	’Lastovka’	1.16
19	’Drobnica’	1.15
71	’Drobnica’	1.15
83	’Drobnica’	1.15
258	’Lastovka’	1.15
312	’Drobnica’	1.14
28	’Drobnica’	1.13
52	’Nocellara’	1.13
55	’Drobnica’	1.13
116	’Drobnica’	1.13
254	’Nocellara’	1.13
158	’Lastovka’	1.05
183	’Lastovka’	1.05
285	’Drobnica’	1.04
289	’Drobnica’	1.02
10	’Drobnica’	0.97
13	’Drobnica’	0.97
23	’Drobnica’	0.97
99	’Drobnica’	0.97
134	’Drobnica’	0.97
140	’Drobnica’	0.97
175	’Lastovka’	0.97

Embryo	Pollen donor	LOD score
182	′Drobnica′	0.95
198	′Lastovka′	0.95
264	′Lastovka′	0.94
148	′Lastovka′	0.93
173	′Lastovka′	0.93
82	′Nocellara′	0.92
280	′Lastovka′	0.90
191	′Lastovka′	0.87
225	′Lastovka′	0.84
193	′Lastovka′	0.83
67	′Pendolino′	0.82
299	′Cempresino′	0.82
147	′Lastovka′	0.81
226	′Lastovka′	0.81
230	′Lastovka′	0.81
261	′Lastovka′	0.81
310	′Lastovka′	0.81
244	′Istarska bjelica′	0.79
16	′Levantinka′	0.77
188	′Lastovka′	0.77
81	′Levantinka′	0.76
105	′Istarska bjelica′	0.72
169	′Lastovka′	0.71
151	′Lastovka′	0.70
17	′Buharica′	0.64
24	′Itrana′	0.64
51	′Buharica′	0.64
54	′Buharica′	0.64
72	′Buharica′	0.64
31	′Nocellara′	0.61
301	′Dužica′	0.61
306	′Dužica′	0.61
159	′Nocellara′	0.60
275	′Lastovka′	0.55
170	′Drobnica′	0.54
279	′Lastovka′	0.51
20	′Leccino′	0.50
87	′Leccino′	0.50
48	′Leccino′	0.48
123	′Leccino′	0.48
212	′Leccino′	0.45
218	′Leccino′	0.45
288	′Leccino′	0.45
142	′Nocellara′	0.44
168	′Drobnica′	0.43
268	′Dužica′	0.42
156	′Leccino′	0.36
308	′Drobnica′	0.30
171	′Mastrinka′	0.26
221	′Dužica′	0.23
77	′Lastovka′	0.22
78	′Lastovka′	0.16
164	′Dužica′	0.09
166	′Drobnica′	0.09
192	′Nocellara′	0.09
242	′Drobnica′	0.05
246	′Cempresino′	0.05
286	′Buharica′	0.03
94	′Levantinka′	0.3
6	No likely pollen donor	
27	No likely pollen donor	
60	No likely pollen donor	
112	No likely pollen donor	
155	No likely pollen donor	
163	No likely pollen donor	
189	No likely pollen donor	
199	No likely pollen donor	
257	No likely pollen donor	

Table S5. Number of embryos from ‘Oblica’ variety assigned to each potential pollen donor in 2018.

	Embryos assigned with only one possible pollen donor	Embryos assigned at LOD score above threshold (1,5)
’Buharica’	8	3
’Cipresino’	6	4
’Coratina’	0	0
**’Drobnica’**	**96**	**91**
**’Dužica’**	**27**	**22**
’Istarska bjelica’	5	3
’Itrana’	2	1
**’Lastovka’**	**133**	**119**
’Leccino’	8	0
’Levantinka’	4	1
’Mastrinka’	1	0
’Nocellara’	10	6
’Oblica’	0	0
’Pendolino’	3	2
Sum	303	252

Table S6. Wind direction (east, E; north, N; northeast, NE; northwest, NW; south, S; southeast, SE; southwest, SW; west, W) and strength (2-5 m/s, light, 1; 5-9.9 m/s, moderate, 2; > 9.9 m/s, strong, 3) during the flowering period in 2017 (light grey) and 2018 (dark grey).

	2017						2018					
	May			June			May			June		
**Days**	**7h**	**14h**	**21 h**	**7 h**	**14 h**	**21 h**	**7h**	**14h**	**21 h**	**7 h**	**14 h**	**21 h**
1.	ENE 2	SW 2	NW 2	E 2	SW 3	E 1	SSW 1	SSW 3	NNE 1	SE 2	WSW 4	NW 4
2.	NNE 2	ESE 2	NW 4	E 1	SW 4	SW 3	E 1	SSW 2	ENE 2	NNE 2	W 3	SSW 4
3.	NNE 2	SSE 1	NW 4	E 2	SW 4	NNW 2	SSW 1	S 4	ESE 3	SE 1	WSW 4	NW 3
4.	E 1	S 2	W 2	E 1	SW 3	W 1	ESE 3	ESE 3	WSW 2	E 2	WSW 3	NNW 2
5.	N 2	W 3	WNW 2	E 2	SW 3	W 2	ESE 2	SW 2	N 1	E 1	SW 3	W 2
6.	E 1	WSW 4	WSW 2	ESE 2	S 2	NW 2	E 2	SW 4	NE 3	E 1	SW 3	N 2
7.	NW 1	WNW 3	S 1	E 1	SW 3	W 2	NE 2	SE 2	NNE 1	ESE 2	WSW 3	NNE 1
8.	NW 3	SW 3	NE 2	ENE 2	WSW 3	ENE 2	C 0	WSW 4	NNW 2	NE 2	ESE 3	NW 2
9.	ENE 2	SW 3	NNE 3	ESE 2	SW 4	NW 1	ENE 1	SSW 2	W 2	NW 4	S 3	SW 1
10.	E 1	SW 3	C 0	NE 1	SSW 3	NW 1	SSW 1	SW 4	WNW 3	SW 2	WNW 3	SSW 2
11.	E 3	ESE 4	E 4	E3	ESE 3	NNE 3	E 2	SW 2	N 2	E 2	SW 3	NNW 2
12.	E 4	ESE 3	N 2	ESE 1	SW 3	WSW 1	E 2	SW 3	SSW 1	E 2	WSW 3	N 2
13.	E 1	SW 4	ENE 2	E 1	SW 4	NNE 1	ENE 2	SW 3	WNW 2	W 2	SW 3	NW 3
14.	E 1	SW 4	WNW 3	SE 1	SSW 3	NE 2	ESE 3	S 4	SSW 2	NE 3	WNW 4	NE 3
15.	E 1	NE 2	NNE 1	NNW 2	ESE 2	WNW 2	E 1	SSE 4	E 2	NE 3	S 2	ENE 3
16.	NE 3	NNE 4	ENE 3	SE 1	SW 4	NE 3	NNE 2	S 2	NNW 2	NW 3	ENE 5	WNW 2
17.	NE 2	NNE 3	N 2	ESE 1	ENE 3	NE 3	E 2	ESE 1	NW 2	N 2	SW 3	NNE 2
18.	E 1	SW 4	NNW 2	N 4	NNE 4	NNW 3	E 1	WNW 3	NW 3	ENE 2	N 3	NNE 4
19.	ESE 2	SW 3	NW 1	ENE 2	SW 4	NW 2	ESE 2	WSW 4	N 2	NNW 2	NE 5	E 3
20.	SW 1	NNW 5	W 2	E 2	SW 3	NE 1	E 2	SW 3	ENE 2	E 1	SW 4	N 2
21.	NW 2	NNE 5	NNW 3	ESE 2	SW 5	N 1	E 2	WSW 3	WSW 2	ENE 2	SSW 4	NE 2
22.	NE 3	SSW 4	NNW 1	NE 1	SSW 3	SW 2	ENE 2	ESE 2	NNE 1	ESE 2	NE 2	NE 4
23.	SSE 1	SW 3	N 2	ESE 2	SW 3	SW 2	NNW 3	SW 4	NW 2	ENE 5	ESE 2	NE 4
24.	NNE 2	E 4	SW 3	ESE 2	SW 3	NW 1	E 2	SW 3	NNW 1	NE 4	SW 3	NNW 2
25.	N 2	ENE 3	NNW 3	E 2	SW 3	WNW 4	SE 1	SW 3	N 2	ENE 3	E 3	NNE 4
26.	NNW 4	NE 4	N 3	E 2	SSW 4	S 2	ESE 2	SW 4	NW 2	E 2	ENE 4	NE 3
27.	NNW 2	ESE 2	N 4	E 2	SSW 3	ESE 2	ESE 1	SW 3	NE 2	NE 4	ENE 3	NNW 3
28.	NNE 3	SW 3	WSW 2	E 3	ESE 2	E 4	SE 2	SW 3	W 2	N3	SSW 2	WNW 4

29.	ENE 2	SW 4	NNW 1	S 3	SW 4	SSW 2	E 2	WSW 3	NW 2	ESE 2	NW 4	WSW 1
30.	E 1	SW 4	SW 1	ESE 3	ESE 3	NW 5	C 0	SW 3	N 2	NW 3	SSW 2	NW 4
31.	ENE 2	SW 3	WSW 2	C 0	C 0	C 0	E 2	SW 4	NNW 1	C 0	C 0	NNW 1
